# Editorial: Advancing knowledge-based economies and societies through AI and optimization: innovations, challenges, and implications

**DOI:** 10.3389/frai.2025.1757072

**Published:** 2026-01-14

**Authors:** Erfan Babaee Tirkolaee, Ramin Ranjbarzadeh, Gerhard-Wilhelm Weber

**Affiliations:** 1 Department of Industrial Engineering, Istinye University, Istanbul, Türkiye; 2 Department of Industrial Engineering and Management, Yuan Ze University, Taoyuan, Taiwan; 3 Department of Mechanics and Mathematics, Western Caspian University, Baku, Azerbaijan; 4 School of Computing, Faculty of Engineering and Computing, Dublin City University, Dublin, Ireland; 5 Faculty of Engineering Management, Poznan University of Technology, Poznan, Poland; 6 Department of Mathematics, METU, Ankara, Türkiye

**Keywords:** Artificial Intelligence–AI, automation, computational intelligence, data-driven analytics, intelligent optimization algorithm, knowledge-based economies and societies

The rapid expansion of Artificial Intelligence (AI), data-driven analytics, and intelligent optimization is reshaping the foundations of modern knowledge-based economies and digitally enabled societies. These developments are no longer confined to laboratories or tech-driven sectors; they are actively transforming industry, governance, education, logistics, and daily social interactions. We are witnessing a fundamental shift toward environments that increasingly rely on Computational Intelligence (CI), automation, and large-scale data interpretation. The research presented in this Research Topic: “*Advancing Knowledge-Based Economies and Societies through AI and Optimization: Innovations, Challenges, and Implications*,” reflects this broader societal transformation. Altogether, the nine contributions highlight emerging opportunities, methodological advancements, and the complex challenges that accompany the deployment of AI and optimization within real socio-economic ecosystems.

Beyond their individual findings, the papers published in this Research Topic together point to a deeper evolution in how AI and optimization are influencing the development of knowledge-based societies. Several contributions converge on the idea that intelligent decision-making, data-driven governance, and socio-economic innovation are becoming tightly interconnected. This trend suggests a future in which knowledge-based economies increasingly depend on autonomous analytical systems, adaptive policy mechanisms, and algorithmic models capable of responding to complex uncertainties. The overall message is clear: AI and optimization are not simply improving existing systems; they are reshaping how knowledge is produced, shared, and applied across institutions, industries, and communities.

At the same time, this rapidly expanding domain faces structural challenges that deserve careful attention. The field continues to struggle with issues such as data quality and accessibility, ethical and transparent AI governance, interpretability of increasingly complex models, and the scalability of optimization techniques in uncertain or data-scarce environments. Methodological gaps persist as well, particularly in integrating domain expertise into Machine Learning (ML)-driven automation and ensuring that technically sophisticated models remain practical for deployment. Yet these challenges also reveal substantial opportunities. Advances in cross-sector data fusion, human-centric algorithm design, and sustainability-oriented optimization approaches represent the emergence of new research pathways with the potential to shape next-generation societal infrastructures. By framing the Research Topic papers against the backdrop of these broader issues, this Editorial spotlights the expanding complexity as well as growing significance of this interdisciplinary field. It sets forth a forward-looking research agenda for scholars and practitioners. The contributions in this Research Topic collectively indicate several priority directions. One urgent need is for more transparent, explainable, and ethically aligned AI systems. Another is the development of optimization models that can operate reliably under real-time uncertainty, especially within dynamic socio-economic environments. A third is the design of socio-technical frameworks that integrate algorithmic insights with human judgment, institutional realities, and societal values. Progress in these areas will require deeply interdisciplinary collaboration, bringing together economists, engineers, computer scientists, policymakers, and social scientists. From an editorial perspective, we argue that future scholarship should move beyond isolated technical improvements and instead work toward scalable, inclusive, and context-aware AI-optimization ecosystems capable of genuinely supporting societal wellbeing. These emerging trajectories form a strategic foundation for guiding the next era of research and practice in knowledge-based economies.

A critical synthesis of the Research Topic contributions reveals meaningful patterns. The papers converge on the recognition that data-driven intelligence, predictive analytics, and decision support tools are becoming foundational across diverse sectors, from public administration and education to manufacturing, transportation, and urban systems. They highlight the need for AI systems that are adaptive, transparent, and responsive to human and societal needs. However, the papers also diverge in their methodological approaches, target domains, and interpretations of socio-technical concerns. These contrasts illuminate persistent points of conflict between technical sophistication and real-world usability, between efficiency-oriented objectives and ethical or equity considerations, and between automation and the preservation of human agency. These patterns also indicate that the field must work toward reconciling such tensions by developing frameworks that balance methodological robustness with relevance to societal needs. For scholars, this means advancing research in areas such as cross-domain modeling, human-AI collaboration, and ethical algorithm design. For practitioners, it emphasizes the importance of implementing AI-optimization systems that are responsible, context-sensitive, and aligned with institutional and community priorities.

Several thematic clusters within the Research Topic further illustrate the transformative role of AI and optimization. One group of papers focuses on AI-powered decision support tools, which are increasingly used to address multifaceted challenges in both public and private sectors. These tools leverage ML, predictive analytics, heuristics, and hybrid models to improve planning, resource management, and organizational adaptability. In knowledge-based societies, where information flows, digital services, and citizen expectations evolve rapidly, such intelligent systems can enhance transparency, accountability, and responsiveness. Another cluster highlights optimization and algorithmic intelligence within industrial and manufacturing contexts. Here, the emphasis lies on improving efficiency, resilience, and sustainability through advanced scheduling, production planning, energy management, and uncertainty modeling. Altogether, these studies show how optimization is becoming a strategic driver of competitive advantage, supporting broader shifts toward digitized, automated, and flexible production ecosystems.

Research on logistics, mobility, and urban systems provides additional evidence of AI's societal influence. The proliferation of urban data, from traffic patterns to environmental indicators, enables the design of more adaptive, responsive, and sustainable mobility solutions. Optimization-driven models help reduce congestion, lower emissions, and improve equitable access to urban services. These studies also bring attention to a crucial point: technological innovation must be paired with human-centered design to ensure real-world impact. Methodological innovations also feature prominently in this Research Topic. Many contributions introduce novel algorithms, improved metaheuristics, hybrid AI-optimization architectures, or specialized modeling tools capable of tackling high-dimensional, nonlinear, or uncertain environments. Importantly, the Research Topic concludes with perspectives on AI in education, knowledge management, and societal development. The contributions remind us that building a knowledge-based society requires not only technological progress but also sustained investment in digital literacy, collaboration, and institutional support.

Finally, we extend our sincere gratitude to all authors who contributed to this Research Topic for their insightful, innovative, and high-quality work. We are equally grateful to the reviewers, whose careful evaluations and constructive feedback significantly strengthened the publications. Our appreciation also goes to the *Frontiers* editorial team for their guidance and continued support throughout the entire process. We hope this Research Topic inspires further investigation at the intersection of AI, optimization, and socio-economic development, encouraging new collaborations and advancing the global dialogue on how technology can responsibly and effectively support knowledge-based societies. As communities, industries, and governments continue to engage with the accelerating pace of digital transformation, the insights collected here offer valuable pathways for shaping more intelligent, sustainable, and inclusive futures.

## Publisher papers

Bélisle-Pipon, Christopher and Nithya, González-Flores et al., Guan et al., Kaplan et al., Lāma and Lastovska, Zhang et al., Schrader et al., Uandykova et al..

